# Combined Yeast Cultivation and Pectin Hydrolysis as an Effective Method of Producing Prebiotic Animal Feed from Sugar Beet Pulp

**DOI:** 10.3390/biom10050724

**Published:** 2020-05-06

**Authors:** Agnieszka Wilkowska, Joanna Berlowska, Adriana Nowak, Ilona Motyl, Aneta Antczak-Chrobot, Maciej Wojtczak, Alina Kunicka-Styczyńska, Michał Binczarski, Piotr Dziugan

**Affiliations:** 1 Institute of Fermentation Technology and Microbiology, Faculty of Biotechnology and Food Science, Łódź University of Technology, Wólczańska 171/173, 90-924 Łódź, Poland; alina.kunicka@p.lodz.pl; 2 Department of Environmental Biotechnology, Faculty of Biotechnology and Food Sciences, Lodz University of Technology, Wólczańska 171/173, 90-924 Łódź, Poland; joanna.berlowska@p.lodz.pl (J.B.); adriana.nowak@p.lodz.pl (A.N.); ilona.motyl@p.lodz.pl (I.M.); piotr.dziugan@p.lodz.pl (P.D.); 3 Institute of Technology and Analysis of Food, Faculty of Biotechnology and Food Science, Lodz University of Technology, Wólczańska 171/173, 90-924 Łódź, Poland; aneta.antczak@p.lodz.pl (A.A.-C.); maciej.wojtczak@p.lodz.pl (M.W.); 4 Institute of General and Ecological Chemistry, Faculty of Chemistry, Lodz University of Technology, Żeromskiego 116, 90-924 Łódź, Poland; michal.binczarski@p.lodz.pl

**Keywords:** pectin-derived oligosaccharides (POS), prebiotics, sugar beet pulp, animal feed

## Abstract

An effective and ecological method for liberation of pectin-derived oligosaccharides (POS) from sugar beet pulp (SBP) was developed using enzymatic and microorganism-mediated biomass conversion. The POS may be applied in the production of prebiotic feed additives. Various yeast strains were screened for their capacity for protein synthesis and monosaccharide assimilation. Combined yeast cultivation and pectin hydrolysis were found to be an effective method of producing prebiotics. Separate enzymatic hydrolysis and fermentation of SBP resulted in the release of 3.6 g of POS per 100 g d.w., whereas the yield of POS acquired after the combined process was 17.9% higher, giving 4.2 g of POS per 100 g d.w. Introducing the yeast into the process improved hydrolysis performance due to lower enzyme inhibition by mono- and disaccharides. The prebiotic effect of the POS was assessed by *in vitro* fermentation using individual cultures of gastrointestinal bacteria. The POS in the SBP hydrolysate effectively promoted the growth of lactobacilli and bifidobacteria. A large increase in adherence to Caco-2 cells in the presence of POS was noted for beneficial *Lactobacillus brevis* strains, whereas pathogenic bacteria and yeast (*C. albicans, C. lusitanie, C. pelliculosa*), responsible for infections in breeding animals, showed much weaker adhesion.

## 1. Introduction

Farm animals, especially in their first few months of life, are exposed to various environmental stresses, often resulting in nutritional problems caused by imbalances in their intestinal consortia of microorganisms. The associated stress leads not only to gastritis, but also affects their immune systems and increases the risk of pathogenic infections. During this period, significant changes in digestive tract microbiome have been observed [1,2]. One of the measures taken to ensure animal welfare is to introduce feed additives such as probiotics, oligosaccharides, enzymes, and organic acids. The use of probiotics offers an alternative to antibiotic therapy, reducing the generation and spread of antibiotic-resistant bacteria in the animal breeding environment, and also the levels of residual antibiotics in milk and meat. Simultaneous administration of probiotics and prebiotics has beneficial effects on fecal microbiota, absorption of feed, animal health, growth, and productivity [3,4]. One of the strategies for improving animal nutrition is feed supplementation with pectic oligosaccharides (POS). These non-digestible oligosaccharides are commonly extracted from a variety of agricultural by-products, including olive pomace, apple pomace, sugar beet pulp (SBP), potato pulp, and citrus peel waste [5]. The role of POS as a prebiotic is to stimulate the growth and activity of probiotic bacteria (e.g., *Lactobacillus* sp., *Bifidobacterium* sp.) and limit the activity of pathogenic and putrefactive bacteria [6,7]. Moreover, one of the products of POS fermentation in the colon is short-chain fatty acids, which inhibit the development of pathogens, increasing the absorption of microelements and modulating the immune system [8]. Sugar beet pulp, a by-product of the sucrose production process used mainly for feeding farm animals, may be a valuable source of POS [5,9]. 

Different approaches have been applied to produce POS from SBP, including chemical methods (processing with externally added acids, hydrothermal treatment) and enzymatic hydrolysis. Enzymatic methods are generally regarded as less efficient. For example, in one study the POS yield from the hydrothermal processing of SBP was around 20% higher than that from enzymatic hydrolysis [10]. Nevertheless, high efficiency is not the only consideration. The enzymatic approach has gained prominence in comparison to hydrothermal and acid hydrolysis processes due to its lower environmental impact and minimal emissions.

The use of microorganisms in biomass conversion processes has been studied extensively. Different processes for hydrolytic and microbial biomass conversion have been applied, including separate hydrolysis and fermentation (SHF), simultaneous saccharification and fermentation (SSF), simultaneous saccharification and cofermentation (SSCF), and consolidated bioprocessing (CBP) [11,12]. However, most of this research proposes valorization of biomass by producing simple sugars, which are then used for the microbial synthesis of different value-added products, such as bioethanol, biofuels, or lactic acid. 

The goal of this study was to develop an effective and ecological method for POS generation using combined yeast cultivation and pectin hydrolysis to produce prebiotic animal feed from sugar beet pulp. The approach is based on a microorganism-mediated process which enhances enzymatic hydrolysis of SBP pectin by obtaining lower enzyme inhibition by mono- and disaccharides, which are immediately consumed by the microorganism. Given the nutritional requirements of young animals, a multistep yeast screening process was performed to maximize the content of valuable microbial protein in feed products. The prebiotic properties of the SBP hydrolysates were evaluated using in vitro tests. 

## 2. Materials and Methods 

### 2.1. Materials

Sugar beet pulp (SBP) was obtained from the Dobrzelin sugar factory KSC SA (Poland) and stored at −20 °C until use. The SBP was hydrolyzed using commercial enzyme preparations: Rohament CL (AB Enzymes) and Rohapect 10L (AB Enzymes).

### 2.2. Biological Material Screened for SBP Fermentation

Yeast strains: *Metschnikowia pulcherrima* NCYC 747, *Scheffersomyces stiptis* NCYC 1541, and *Kluyveromyces marxianus* NCYC 179 were acquired from National Collection of Yeast Culture (UK). *Saccharomyces cerevisiae* TT ŁOCK105, *Saccharomyces cerevisiae* Tokay ŁOCK 0204, and *Kluyveromyces lactis* ŁOCK 0028 were acquired from the collection at the Institute of Fermentation Technology and Microbiology (ŁOCK 105), Łódź University of Technology, Poland. Two yeast strains were isolated from commercial preparations: *Saccharomyces cerevisiae* Ethanol Red, Leaf/Lesaffre Advanced Fermentation, and *Saccharomyces cerevisiae*, Lalvin ICV K1-V1116 Lallemand. *Metschnikowia sinensis* 1.4 TKC was isolated from the environment.

Strains of lactic acid bacteria (LAB): seven strains of *Lactobacillus* sp. bacteria were acquired from the collection at the Institute of Fermentation Technology and Microbiology (ŁOCK 105): *Lactobacillus rhamnosus* ŁOCK 0900, *Lactobacillus brevis* ŁOCK 0912, *Lactobacillus casei* ŁOCK 0924, *Lactobacillus brevis* ŁOCK 0983, *Lactobacillus plantarum* ŁOCK 0990, *Lactobacillus plantarum* ŁOCK 0991, and *Lactobacillus paracasei* ŁOCK 0993. *Lactobacillus plantarum* ATCC 8014 was sourced from the ATCC collection (American Type Culture Collection). The biological material included strains isolated from the environment, which were identified by molecular methods based on 16S rRNA gene sequencing and deposited in the NCBI GenBank database. These included *Lactobacillus plantarum* AXD KT751284, *Lactobacillus plantarum* AXG KT751285, and Jerusalem artichoke silage isolates of *Leuconostoc mesenteroides* (strain number 5, 6, 7), as well as four isolates from beet pulp silage: *Lactobacillus brevis* W13A, *Lactobacillus plantarum* W12, *Lactobacillus plantarum* W12A, and *Lactobacillus brevis* W8A. 

The strains were stored in Cryobanks™ (Copan Diagnostics Inc., USA) at a temperature of −22 °C. Before the experiments, the lactic acid strains were activated and passaged twice in de Man, Rogosa, and Sharpe broth (MRS; Merck, Darmstadt, Germany) for 24 h at 37 °C. The yeast was grown on yeast extract, peptone, glucose (YPG) for 24 h in 25 °C. 

### 2.3. Yeast Screening

#### 2.3.1. Cell Concentration of Yeast Strains

Yeast were cultivated in aerobic conditions at 25 °C for 24 h in two different media based on SBP hydrolysates: Medium A containing SBP hydrolysate only, and Medium B containing SBP hydrolysate enriched with anhydrous di-potassium hydrogen phosphate and anhydrous di-ammonium hydrogen phosphate, each added at 0.3 g/L, which were used as supplements. The SBP hydrolysate was prepared by enzymatic hydrolysis, which was performed according to the method described in Section 2.4, except that for the purposes of screening, yeast cultivation was performed after enzymatic hydrolysis, as a sequential rather than simultaneous process. The yeast cell concentration (cfu/mL) was determined using the plating method on YPG medium. The petri plates were incubated at 25 °C for 24 h.

#### 2.3.2. Utilization of Monosaccharides 

Monosaccharides (glucose, fructose, mannose, galactose, arabinose, xylose) were determined by UV spectrophotomety using Megazyme (Wicklow, Ireland) enzymatic kit tests: the D-Mannose/D-Fructose/D-Glucose Assay kit (K-MANGL), L-Arabinose/D-Galactose Assay Kit (K-ARGA); and the D-Xylose Assay Kit (K-XYLOSE). The assays were performed using a Multiscan GO spectrophotometer (Thermo Scientific) equipped with a microplate reader. The monosaccharide concentrations were determined immediately before yeast inoculation on SBP and again after 48 h of incubation.

#### 2.3.3. Protein Concentration

The yeast present in the SBP preparations after SHF were submitted to autolysis process. Autolysis was performed at 55 °C for 48 h. The soluble protein content in the SBP preparations was analyzed using a Direct Detect® System (Merck-Millipore, Massachusetts, USA). This method is based on Fourier transform infrared spectroscopy, which detects amide bonds in the protein structure.

Total nitrogen was analyzed using the Kjeldahl procedure after the SSF process. The sample (1 g) was digested in concentrated sulfuric acid (10 mL) in the presence of VCT catalyst tabs (VELP Scientifica) under the following temperature conditions: 150 °C (60 min), 200 °C (120 min), 300 °C (40 min), 420 °C (60 min). After mineralization, the ammonia was steam distilled into boric acid solution (2% v/v) under alkaline conditions and then estimated by hydrochloric acid titration. On the basis of the nitrogen content, the amount of crude protein in the sample was calculated.

#### 2.3.4. Antagonistic Properties of Yeast Metabolites against Lactic Acid Bacteria

The disk diffusion method (Kirby–Bauer test) was used to determine the antagonistic properties of yeast strains metabolites against strains of LAB. The metabolite diffusion test was conducted using YPG agar disks containing submerged yeast cells. To determine the sensitivity of the LAB bacteria to yeast metabolites, the disks were placed on an agar plate where the bacteria were inoculated. The zones of inhibited bacterial growth around the disks (excluding the diameter of the disk) were measured after 48 h of incubation at 37 °C under anaerobic conditions. 

### 2.4. SSF Process

The SBP was blended with water in proportions of 1:3 (w/w) and homogenized to obtain 0.8–1.0 mm particles. The pH was adjusted to 5.0 using 0.1 M HCl. The cellulolytic enzyme Rohament CL (AB Enzymes) and the pectinolytic enzyme Rohapect 10L (AB Enzymes) were added simultaneously to the SBP preparation, at doses of 150 ppm and 1500 ppm, respectively [13]. The SBP was supplemented with anhydrous di-potassium hydrogen phosphate and anhydrous di-ammonium hydrogen phosphate, each added at 0.3 g/L, and inoculated with 3% yeast culture. The process of simultaneous enzymatic hydrolysis and yeast breeding (in aerobic conditions) was carried out in a Parr Instrument Company model 4552 reactor with a working volume of 5 L, equipped with a temperature control and mixing system (500 rpm). Samples of 3 L of SBP were processed at 25 °C for 24 h, after which the enzymes were inactivated by denaturation performed at 85 °C for 10 min. The samples were collected to determine the POS and protein concentration. The SBP specimen was also assayed for its prebiotic activity by evaluating its influence on gastrointestinal microbiota and antiadhesive activity.

### 2.5. High Performance Anion Exchange Chromatography

Chromatographic analysis of pectin-derived oligosaccharides was performed according to the method developed in our previous study [13], using a DIONEX ICS-3000 ion chromatograph (Dionex, USA) with an electrochemical PED detector, equipped with a Dionex Carbo Pac PA200 (3 × 250 mm) analytical column and a Dionex Carbo Pac PA200 guard column (3 × 50 mm). Gradient separation was used at a 0.8 mL/min flow rate with the following eluents: Eluent 1: 200 mM NaOH (solution 50% v/v in water NaOH, Sigma-Aldrich®, Europe) in 550 mM NaOAc (Sigma-Aldrich®, Europe); eluent 2: 250 mM NaOH (solution 50% v/v in water NaOH, Sigma- Aldrich®, Europe); eluent 3: distilled water (18MΩ). The oligomer standards were obtained according to the procedure described in [14].

### 2.6. In Vitro Fermentability of POS

Bacterial strains abbreviated as ŁOCK were acquired from the Collection of the Institute of Fermentation Technology and Microbiology (ŁOCK 105), Łódź University of Technology, Poland. Bacterial strains abbreviated as ATCC were purchased from the American Type Culture Collection. The following strains were used: *Lactobacillus* sp. (*Lb. plantarum* ŁOCK 0995, *Lb. plantarum* ŁOCK 0981, *Lb. plantarum* ŁOCK 0989, *Lb. brevis* ŁOCK 0984); *Bifidobacterium* sp. (*Bifidobacterium 2, Bifidobacterium 3* – strains isolated from the human feces) and pathogens (*Escherichia coli* ATCC 10536, *Escherichia coli* ATCC 8739, *Salmonella* Typhimurium ATCC 14028, *Listeria monocytogenes* ŁOCK 18195, *Staphylococcus aureus* ATCC 6538). 

To study the growth of individual bacterial strains in the presence of POS, the cultures were seeded on a sterile SBP hydrolysate using 2% of inoculum and incubated at 37 °C for 21 days (pH = 7 ± 0.1). The microorganisms were incubated at 37 °C for 48 h with the application of a suitable agar medium (*Lactobacillus* sp. – De Man Rogosa and Sharpe (MRS) Agar (Merck, Darmstadt, Germany), *Bifidobacterium* sp.—Bifidobacterium medium; pathogenic bacteria – Agar TSA (Merck, Darmstadt, Germany), and counted.

### 2.7. Adhesion of Lactic Acid Bacteria (LAB) or Pathogens to Human Gut Epithelial Cells

#### 2.7.1. Bacterial Strains

The following strains of LAB (of *Lactobacillus* sp.: *Lb. rhamnosus* ŁOCK 0900, *Lb. plantarum* ŁOCK 0991, *Lb. brevis* ŁOCK 0983 and ŁOCK 0984), pathogenic bacteria (*Escherichia coli* ATCC 8739 and ATCC 10536, *Staphylococcus aureus* ATCC 6538 and ATCC 25923, *Klebsiella aerogenes* ATCC 13048 and *Enterococcus faecalis* ATCC 29212), and *Candida* sp. yeast strains (*C. albicans* ATCC 10231, *C. lusitaniae* ŁOCK 0004, *C. utilis* ŁOCK 0021, and *C. pelliculosa* ŁOCK 0007) were used. In the adherence assay, lactobacilli were cultured on De Man Rogosa and Sharpe (MRS) Agar (Merck, Darmstadt, Germany), bacterial pathogens on Plate Count Agar (Merck, Darmstadt, Germany), and yeast on YPG (yeast extract, peptone, glucose) with agar.

#### 2.7.2. Caco-2 Cell Culture

Caco-2 cells (Cell Lines Services, Eppelheim, Germany) were cultured in T75 Roux bottles, as described in detail elsewhere [13]. Briefly, the cells were cultured in Dulbecco Modified Eagle’s Medium (DMEM, Sigma-Aldrich, St. Louis, MO, USA), supplemented with 4 mM GlutaMAXTM (Thermo Fisher Scientific, Waltham, MA, USA), 25 mM HEPES buffer (Sigma-Aldrich, St. Louis, MO, USA), 10% fetal bovine serum (FBS, Thermo Fisher Scientific, Waltham, MA, USA), and 100 µg/mL streptomycin/100 IU/mL penicillin mixture (Sigma-Aldrich, St. Louis, MO, USA) for 5 days at 37 °C in an atmosphere of 5% CO_2_. Every 3 days, the cells were washed with phosphate buffer saline (PBS, pH 7.2, Sigma-Aldrich, St. Louis, MO, USA) and medium was changed. Confluent cells were detached using TrypLE^TM^ Express (Thermo Fisher Scientific, Waltham, MA, USA), according to the manufacturer’s instructions. A suspension of the cells was centrifuged (182 × g, 5 min) and decanted. The pellet was re-suspended in fresh DMEM. Cell viability was determined by trypan blue exclusion in a hemocytometer.

#### 2.7.3. Adherence Assay

The adherence assay was conducted according to a process described previously [13]. Caco-2 cells were seeded into a 24-well plate at a concentration of 2.5 × 10^5^ cells/well and left overnight. All microorganisms were cultured for 24 h at 30 or 37 °C. The cultures of Lactobacillus sp. were diluted in sterile MRS to bring the number of bacteria to 3.5 × 10^9^ CFU/mL (initial number). This level was estimated spectrophotometrically from the standard curves. The initial number of pathogenic bacteria and yeasts (10^8^ CFU/mL) was determined using a DEN-1 McFarland densitometer (Biosan, Riga, Latvia). The microorganisms were then centrifuged, the supernatants were removed, and the pellets were washed with sterile PBS and centrifuged again to unload the residual substrate. Enzymatic hydrolysate of beetroot pulp was added to the bacterial pellets, while to the control sample DMEM was added without supplements. Next, the medium was removed from the 24-well plate and 1 mL of the suspension of microorganisms was added to the Caco-2 cells in in three replicates. The plate was incubated for 2 h at 37 °C in 5% CO_2_ with humidity > 95%. Non-adhered microorganisms were removed by double washing with PBS. To detach the Caco-2 cells with adhered bacteria, 1% trypsin (Sigma-Aldrich, St. Louis, MO, USA) was added to each well and the samples were incubated for 10 min at 37 °C. Detachment was observed under an inverted microscope. Next, the cells with adhered bacteria (in PBS) were transferred into sterile Eppendorf tubes, centrifuged, and decanted. To lyse the Caco-2 cells and prevent the formation of microbial aggregates, the resulting pellets were re-suspended in 0.1% Triton X-100 and incubated for 5 min at room temperature. The numbers of microorganisms which had adhered to the Caco-2 cells were counted using Koch’s plate method, with the application of a suitable agar medium, and incubated at 37 °C for 48 h. 

The adherence rate was calculated as follows:

AR = [logA_0_/logA_1_] × 100% (1)

where A_0_ means log CFU of the initial bacteria added to the well and A_1_ is log CFU of the adhered bacteria. 

The stimulation/inhibition rate [%] was calculated as follows:

SR/IR = [A_T_ × 100/A_0_] − 100 (2)

where A_T_ means adherence of tested sample and A_0_ is adherence of control. This gave the percentage increase/reduction in adhesion for the tested microorganisms in the presence of beetroot pulp hydrolysate.

Microscopic observations were performed on 4-well Lab-TekTM slides (Nunc, Thermo Fisher Scientific, Waltham, MA, USA), according to the method described above. After 2 h of incubation with beetroot pulp hydrolysate on the monolayer of the Caco-2 cells, the non-adhered bacteria were removed. The wells were washed with PBS and fixed with 70% methanol (15 min, ambient temperature), stained with 0.5% crystal violet, washed with 70% ethanol and dried overnight. Adhered microorganisms were observed at 400× magnification under a microscope (Nikon, Tokyo, Japan) connected to a camera (Nikon Digital Sight DS-U3, Nikon, Tokyo, Japan) using imaging software (NIS-elements BR 3.0, Nikon, Tokyo, Japan).

### 2.8. Statistical Analysis

The data were analyzed using two-way analysis of variance (ANOVA). The differences between samples with normal distributions were evaluated using a Tukey test. Both the Tukey test and ANOVA were performed using SPSS software (IBM). Significant differences were accepted at p < 0.05. The results are presented as the mean ± SD. All experiments were performed in triplicate.

## 3. Results and Discussion

### 3.1. Biomass Productivity and Profile of Monosaccharides in SBP Hydrolysates utilized by Yeast 

**Figure 1:**
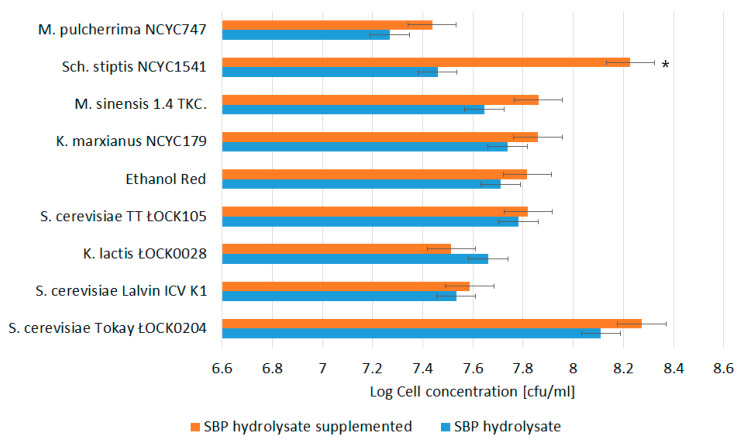
Yield of yeast cells cultivated on sugar beet pulp (SBP) hydrolysate. The data represent means from three replicates in one experiment. Error bars denote SD. * Results significantly different from unsupplemented control, ANOVA (*p* < 0.05).

Various yeast strains belonging to the genera *Metschnikowia, Scheffersomyces, Kluyveromyces*, and *Saccharomyces* were seeded on media prepared on the basis of SBP hydrolysates with (B medium) or without (A medium) nitrogen supplementation. The largest numbers of colony forming units per 1 mL of hydrolysate were recorded for the *S. cerevisiae* Tokay ŁOCK 0204 strain in both tested media (1.3·10^8^ in A and 1.9·10^8^ in B medium, respectively). This strain was also characterized by the largest cell concentration increase on both substrates (Figure 1). Nitrogen supplementation did not clearly improve cell yield. Only in the case of *Sch. stiptis* NCYC 1541 was a significant difference (*p* ˂ 0.05) in cell yield noted between A and B media (a 5.9-fold increase). According to Patelski et al. [15], the ability to absorb di-ammonium hydrogen phosphate depends on the conditions of yeast breeding, the composition of the medium, and the enzymatic apparatus of the given strain (strains within one species may differ in their ability to assimilate (NH_4_)_2_HPO_4_). Patelski et al. [15] report that *S. cerevisiae* yeast are capable of absorbing di-ammonium hydrogen phosphate as a nitrogen source, resulting in increased biomass growth. This finding is not supported by the results obtained in our study.

**Figure 2:**
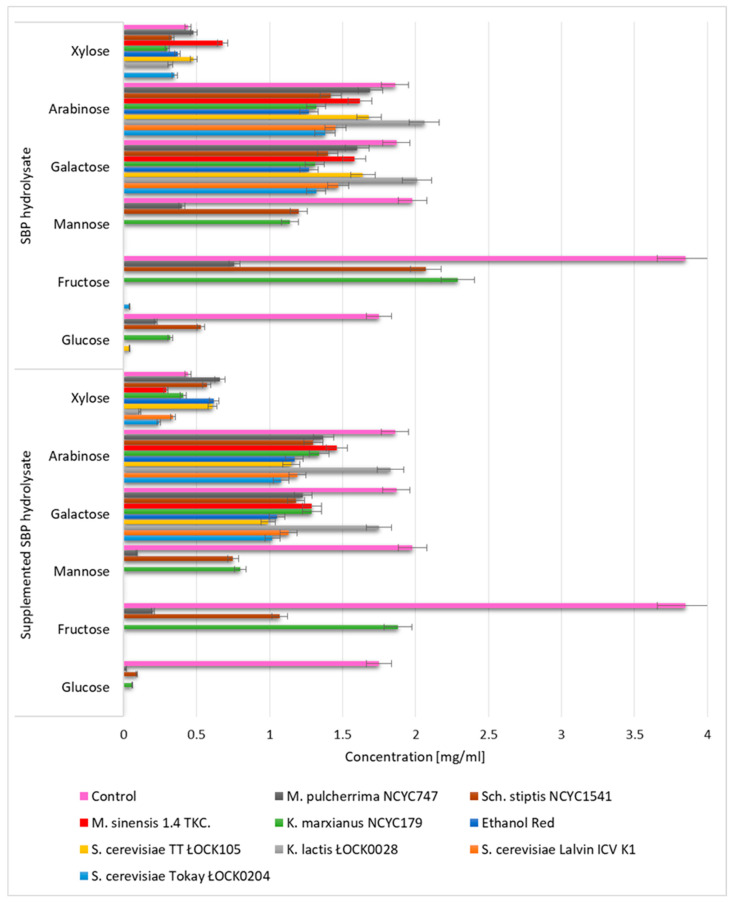
Monosaccharide utilization by different yeast species cultivated in SBP hydrolysate. The data represent means from three replicates in one experiment. Error bars denote SD.

We next evaluated the ability of various yeast species to assimilate the monosaccharides present in SBP hydrolysates (Figure 2). Monosaccharide concentrations were determined before inoculation of the media and after 24 h of breeding. During yeast cultivation on SBP hydrolysate, the glucose, fructose, and mannose were utilized completely or to a great extent by six of the nine tested strains *(S. cerevisiae* TT ŁOCK 105, *S. cerevisiae* Tokay ŁOCK0 204, *K. lactis* ŁOCK 0028, *S. cerevisiae* Ethanol Red, *S. cerevisiae Lalvin*, *M. sinensis* 1.4 TKC). The addition of supplements led to a clear improvement in the assimilation of monosaccharides by these yeasts. The lowest consumption of galactose, arabinose, and rafinose was recorded for *K. lactis* ŁOCK 0028, whereas the highest was noted for all tested *Saccharomyces* strains. P. Patelski et al. [15] report that yeast from the species *S. cerevisiae* consume large proportions of the glucose and fructose contained in hydrolyzed beet pulp, with little use of xylose, galactose, and arabinose. The xylose concentrations increased in the *M. pulcherrima* NCYC 747, *Sch. stiptis* NCYC 1541, and *M. sinensis* 1.4 TKC cultivars. This may have been due to the ability of these yeast strains to release mono-, and disaccharides from oligosaccharides and polysaccharides present in the hydrolyzate. Our results indicate that the examined yeast strains may be successfully used to remove glucose, fructose, and mannose from SBP hydrolysates. Low levels of galactose and arabinose consumption by yeast may be advantageous for the parallel or subsequent culture of probiotic bacteria. Proper selection of yeast may contribute to develop a synbiotic feed on the basis of the prebiotic preparation obtained in this study from sugar beet pulp.

The cultures of the yeast strains in the hydrolyzate were autolyzed to release the protein. The largest amount of soluble protein was found in SBP cultivated with *S. cerevisiae* Tokay ŁOCK 0204, which had the highest cell yield (Table 1). The lowest amounts of protein were found in the yeast autolysates of *Sch. stiptis* NCYC 1541—(0.086 ± 0.011) mg/ml, *K. lactis* ŁOCK 0028—(0.53 ± 0.050) mg/mL, and *S. cerevisiae* TT ŁOCK 105, in which no protein was detected. Alexandre et al. [16] reports that individual strains might be more or less susceptible to autolysis, which does not mean that a strain that autolyzes less efficiently is less valuable as a source of SCP.

The antagonistic properties of yeast metabolites were also evaluated against LAB. The intestine of a newly born animal is sterile. The intestinal bacterial communities undergo dynamic changes during the first weeks of life. The secretion of yeast metabolites inhibiting the growth of LAB may limit the use of these microorganisms as a feed component due to their possible negative impact on the balance of microbiota during colon colonization, especially on valuable probiotic bacteria. The strongest antagonistic activity, expressed as the diameter of the inhibition zone, exerted against the tested LAB were as follows: for *M. pulcherrima* NCYC 747 against *Lb. brevis* ŁOCK 0983 (1.3 ± 0.59 mm) and against *Lb. brevis* W8A (4.0 ± 1.00 mm); for *M. sinensis* 1.4 TKC against *Lb. brevis* W8A (1.0 ± 0.01 mm); for *K. lactis* ŁOCK 0028 against *Lb. brevis* ŁOCK 0983 (1.0 ± 0.03 mm), and for *S. cerevisiae* TT ŁOCK 105 against *Lb. plantarum* AXD (0.3 ± 0.63 mm). Therefore, the tested yeast strains cannot be recommended for the production of prebiotic animal feed.

### 3.2. Yield of Oligosaccharides with Different Degrees of Polymerization Obtained by Separate or Simultaneous Enzymatic Hydrolysis and Production of SCP from Sugar Beet Pulp 

Two different strategies for enzyme and microorganism-mediated SBP biomass conversion were explored in this study: separate hydrolysis and yeast cultivation, and simultaneous hydrolysis and yeast cultivation. At this stage of the investigation, we focused on whether the use of simultaneous hydrolysis and yeast cultivation would influence the release of POS from SBP pectin due to lower enzyme inhibition by mono- and disaccharides, which were immediately consumed by the microorganism. Based on our prior research, *S. cerevisiae* Tokay ŁOCK0204 was selected for its monosaccharide utilization profile and protein production. This strain also exhibits no antagonistic properties against LAB, which may be relevant for maintaining balance in the gastrointestinal tract microbiota of young animals. Enzymatic hydrolysis of SBP was performed for 24 h using a mixture of commercial cellulase (Rohament Cl, AB Enzymes) and pectinase preparation (Rohapect 10L, AB Enzymes). 

**Figure 3:**
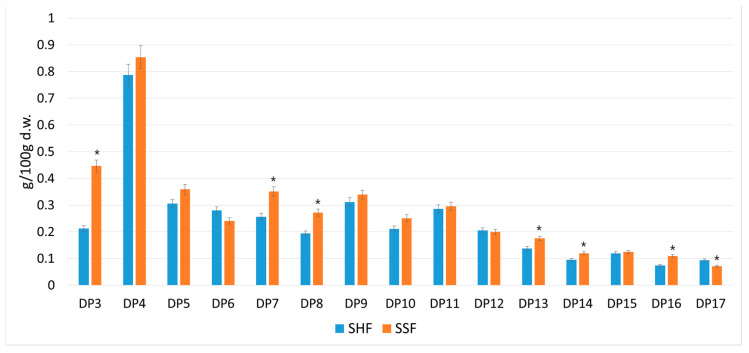
Yield of oligosaccharides with different degrees of polymerization DP3‒DP17 obtained in the process of separate enzymatic hydrolysis and yeast cultivation (SHF), and simultaneous enzymatic hydrolysis and biomass cultivation (SSF). The data represent means from three replicates in one experiment. Error bars denote SD. * Results significantly different from control, ANOVA (*p* < 0.05).

As shown in Figure 3, chromatographic analysis confirmed the presence of a mixture of oligosaccharides with different degrees of polymerization, in the range of DP3–DP17, in both tested samples. The results show that efficient simultaneous hydrolysis and microbial conversion of SBP was achieved. Introducing the yeast into the process caused a decrease in the concentration of digalacturonic acid by 9.3%, which further improved the hydrolysis performance. Pure enzymatic hydrolysis of SBP resulted in the release of 3.6 g of POS per 100 g d.w., whereas the yield of POS acquired from the simultaneous enzyme and microorganism-mediated process was 17.9% higher, giving 4.2 g of POS per 100 g d.w. Combining of both biotechnological processes eliminated the time needed for separate hydrolysis of the pectin present in SBP. Consequently, it reduced the total time required for the process, from preparation of the SBP medium for yeast cultivation to obtaining the final product.

Enzymatic methods of POS production are generally regarded to be less efficient. According to Gonzalez-Garcia et. al. [10] the POS yield of the autohydrolysis of SBP (hydrothermal processing at 163 °C) was around 20% higher than that from an enzymatic process (hydrolysis of SBP performed by a combined enzyme concentrate of Cellulast and Viscozyme at 37 °C). The results obtained in our work clearly show that enzymatic hydrolysis combined with yeast cultivation generates similar levels of POS yield to chemical processing, while avoiding additional energy consumption and the generation of new waste during the extraction of prebiotics from the post-reaction medium.

Usually, the use of simultaneous saccharification and fermentation of SBP with yeast or bacteria cultures requires total decomposition of SBP polysaccharides. The main products, e.g., lactic acid or ethanol, are then synthetized by microorganisms that utilize the released monosaccharides [17,18]. Our approach in this study was different. The final product, prebiotic oligosaccharides, was not generated by microorganisms but during the hydrolytic process. The main function of the yeast was to ensure the proper conditions for obtaining high enzymatic activity. The amount of protein content was 16.4 g/100 g d.w. and 13.2 g/100 g d.w. for the use of SSF and SHF process, respectively.

### 3.3. Influence of SBP Animal Feed Component on the Growth of Selected Lactic Acid Bacteria (LAB) or Pathogens

Currently, probiotic and prebiotic preparations are standard in many milk substitutes for newborn farm animals with unstable gastrointestinal tract microbiomes. The balance of microorganisms colonizing the digestive tract is especially disturbed by the stress conditions to which young animals are exposed (separation from the mother, change of feed, transport, improper zoohygienic conditions). This leads to an increase in the susceptibility of young animals to pathogen colonization and subsequent diarrhea or respiratory disease. It has been suggested that several oligosaccharides (fructooligosaccharides [19,20], mannan oligosaccharides [21,22,23], cellooligosaccharides [24,25], and inulin and lactulose [26,27]) may have functionalities that could improve young animals performance and health. However, modifications of gastrointestinal tract microbiomes by these sugars have yet to be examined in detail.

**Figure 4:**
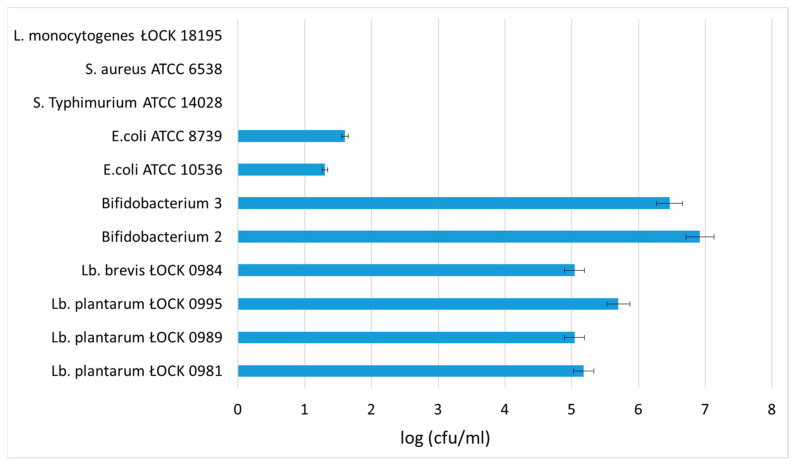
Cell growth of intestinal bacteria species determined after *in vitro* fermentation in SBP hydrolysate. The data represent means from three replicates in one experiment. Error bars denote SD. * Results significantly different from the control, ANOVA (*p* < 0.05).

In this study, the prebiotic potential of pectin-derived oligosaccharides was assessed in terms of its ability to support the growth of selected beneficial bacteria typical for the intestinal microbiota, as well as major enteric pathogens known to cause diarrhea in animals. *In vitro* fermentations confirmed that the POS contained in the SBP hydrolysate were utilized by all tested strains of bifidobacteria and lactobacilli. Intense growth of *Bifidobacterium* sp. (2.9·10^6^ and 8.3·10^6^ cfu/mL) as well as *Lactobacillus* sp. (ranging from 1.1·10^5^ cfu/mL to 5.0·10^6^ cfu/mL) was observed after 21 days of fermentation. On the other hand, the POS were not fermented by *S.* Typhimurium ATCC 14028, *S. aureus* ATCC 6538, or *L. monocytogenes* ŁOCK 18195. Growth of *E.coli* ATCC 10536 and *E.coli* ATCC 8739 was scarcely supported by the SBP hydrolysate (Figure 4). The currently accepted criterion for prebiotic activity is an increase in bifidobacteria and lactobacilli, with a decrease in less desirable bacteria [28].

A stable microbial load of *Lactobacillus* sp. has been shown to improve weight gain and immunocompetence in young calves [29], goat kids [30], and pigs [31]. Carbohydrates are hardly digested by non-ruminants (including preruminant calves), as they resist enzyme degradation, reaching the intestines intact to exert their functions.

### 3.4. Influence of the SBP Animal Feed Component on the Adhesion of Lactic Acid Bacteria (LAB) or Pathogens to Caco-2 Cells

**Figure 5:**
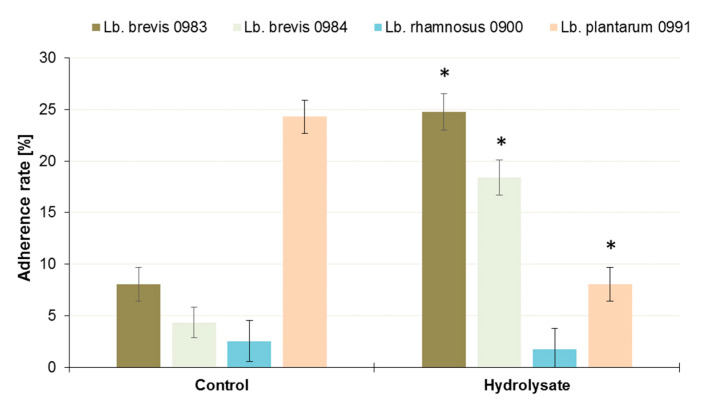
Adherence of *Lactobacillus* sp. strains to Caco-2 cell monolayers in the presence of SBP hydrolysate relative to the control samples. The data represent means from three replicates in one experiment. Error bars denote SD. * Results significantly differ from the relevant control, ANOVA (*p* < 0.05).

The prebiotic properties of the POS contained in the SBP hydrolysate were assessed by evaluating its influence on the adhesive properties of selected LAB (often recognized as probiotic with pro-health properties), as well as against pathogenic bacteria and yeast, which are often sources of infections in humans and breeding animals. Of the *Lactobacillus* sp. bacteria, two *Lb. brevis strains*, ŁOCK 0983 and 0984a, showed a dramatic increase (*p* < 0.05) in adherence to Caco-2 cells in the presence of SBP hydrolysate (Figure 5), and stimulation of adherence of over 200% and 300%, respectively (Table 2). The strain *Lb. rhamnosus* ŁOCK 0900 was weakly adhesive, and in the presence of the hydrolysate, slight inhibition of adherence was observed (30.7%). Almost 70% inhibition was observed in the case of *Lb. plantarum* ŁOCK 0991 (Figure 5, Table 2). The influence of the tested hydrolysate on the adhesive properties of selected *Lactobacillus* sp. bacteria was therefore strain specific.

**Figure 6:**
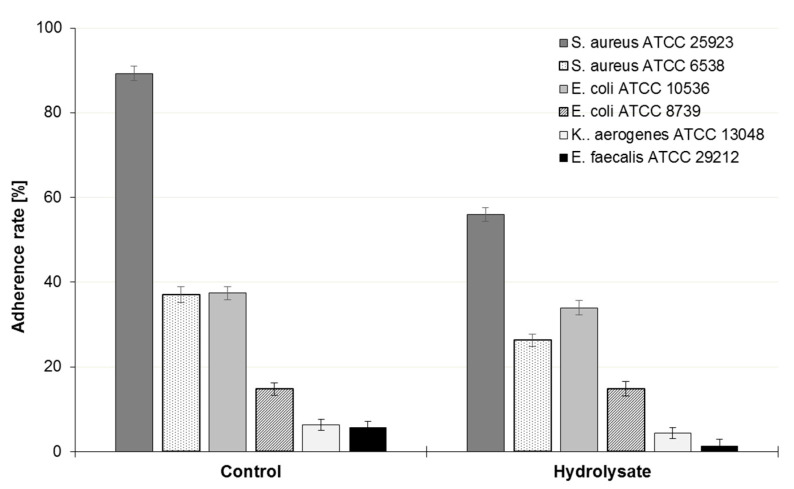
Adherence of pathogenic bacteria to Caco-2 cell line in the presence of SBP hydrolysate relative to the control samples. Error bars denote SD. The data represent means from three replicates in one experiment. The results do not differ significantly from the unexposed controls, ANOVA (*p* < 0.05).

The adherence of almost all pathogenic bacteria was inhibited by the tested SBP hydrolysate, but the inhibition was not statistically significant (*p* < 0.05) (Figure 6, Table 2). The adherence rate of *S. aureus* ATCC 25923 in the control was close to 90%, while in the presence of hydrolysate it decreased to 56% (Figure 6), with an inhibition rate of 30%. The adherence of both *E coli* strains was similar in the controls and in the presence of hydrolysate. The adherence of *E. faecalis* ATCC 29212 was inhibited by 77.8%.

**Figure 7:**
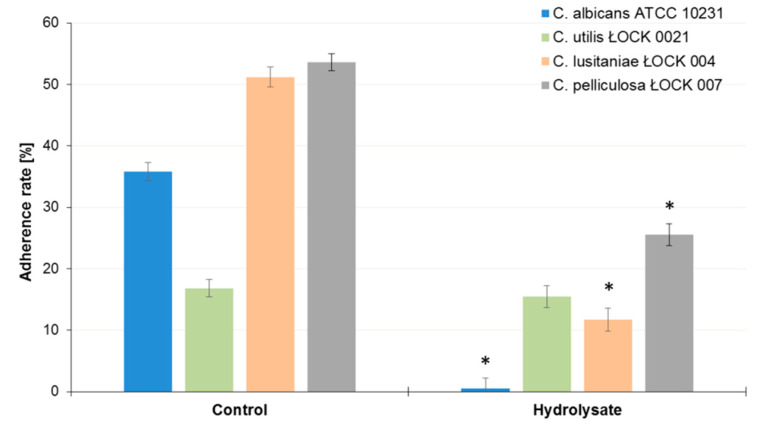
Adherence of Candida sp. yeast to Caco-2 cells in the presence SBP hydrolysate in comparison to the control samples. Error bars denote SD. The data represent means from three replicates in one experiment. * Results significantly differ from the relevant control, ANOVA (*p* < 0.05).

The SBP hydrolysate inhibited adherence by all tested *Candida* sp. strains (Figure 7). The inhibition rate was the strongest for *C. albicans* ATCC 10231 (98.5%, *p* < 0.05), while it was the weakest for *C. utilis* ŁOCK0021 (7.8%) (Table 1). For *C. lusitanie* ŁOCK 0004 and *C. pelliculosa* ŁOCK 0007, the inhibition rates were 77.1% and 52.5%, respectively.

**Figure 8:**
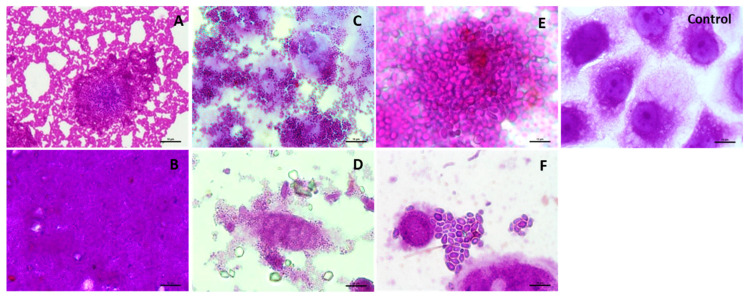
Selected microphotographs showing adherence to Caco-2 cells of: *L. brevis* ŁOCK 0983–without (**A**) and with (**B**) hydrolysate; *S. aureus* ATCC 25923–without (**C**) and with (**D**) hydrolysate; *C. albicans* ATCC 10231 without (**E**) and with (**F**) hydrolysate (× 400, Nikon Eclipse, Japan).

The tested SBP hydrolysate therefore inhibited the adherence of all tested pathogens to Caco-2 cells, in particular in the case of *C. albicans* ATCC 10231 (inhibition rate 98.5%). The hydrolysate also strongly stimulated the adherence of selected beneficial bacteria (*Lb. brevis* ŁOCK 0983 and 0984). Figure 8 shows microphotographs of adherence to Caco-2 cell monolayers by selected microorganisms in the presence and without the tested SBP hydrolysate.

## 4. Conclusions

The beneficial effects of probiotics, prebiotics, and symbiotic preparations on the gastrointestinal tract have made them particularly useful for feeding young animals and improving animal rearing rates. Such preparations are particularly recommended in the cases of young animals exposed to stress and convalescents. The market for probiotics and prebiotics is considerable and growing, due to the declining acceptance of antibiotics in animal feeds and milk substitutes. This paper describes a novel approach to the generation of POS derived from sugar beet pulp for use as prebiotics for farm animals. In particular, it has been shown that the SSF process of POS production is more efficient than the SHF process. The approach was based on a microorganism-mediated process that enhances enzymatic hydrolysis of SBP pectin by obtaining lower enzyme inhibition by mono- and disaccharides, which are immediately consumed by the microorganism. Conventional *Saccharomyces* and non-*Saccharomyces* yeasts were used for enzymatic and microorganism-mediated conversion of POS from SBP. The results of our work clearly show that enzymatic hydrolysis combined with yeast cultivation generates similar levels of POS yield to chemical processing, while avoiding additional energy consumption and the generation of new waste during the extraction of prebiotics from the post-reaction medium. Furthermore, the POS produced from this process exerted the desired prebiotic effect in *in vitro* models, and therefore have potential to be used as prebiotics for farm animals.

Due to its low cost and ease of acquisition, sugar beet pulp can compete with other raw materials used for the synthesis of prebiotics in the form of oligosaccharides. Unlike other methods of producing prebiotics, the technology proposed in this paper for the production of prebiotic feed supplements from SBP is waste-free. The prebiotics can be extracted from the post-reaction medium without the generation of new waste. This innovative technology therefore has the potential to not only increase the yield and profitability of the process but also reduce its environmental impact. It could therefore contribute to current progress towards the circular economy, improving product lifecycles through better recycling and conversion of low-value side streams into more valuable products.

## Figures and Tables

**Table 1:** Protein content in hydrolysate fermented using different yeast strains.

Yeast Strain	Protein Content [mg/mL]
*S. cerevisiae* Tokay ŁOCK0204	0.41 ± 0.07^a^
*S. cerevisiae* Ethanol Red	0.16 ± 0.02^b^
*S. cerevisiae* Lalvin ICV K1	0.29 ± 0.06^b^
*M. pulcherrima* NCYC747	0.22 ± 0.02^b^
*M. sinensis* 1.4 TKC.	0.20 ± 0.09^b^
*Sch. stiptis* NCYC1541	0.09 ± 0.01^c^
*K. marxianus* NCYC179	0.11 ± 0.07^c^
*K. lactis* ŁOCK0028	0.05^c^

Different letters (a, b, c) represent significance difference (*p* < 0.05).

**Table 2:** Stimulation/inhibition of adherence to Caco-2 cell monolayers for tested microbial strains in the presence of SBP hydrolysate.

Strain	Stimulation (+) or Inhibition Rate (-) [%]
*Lb. brevis* 0983	+206.8
*Lb. brevis* 0984	+324.8
*Lb. rhamnosus* 0900	−30.7
*Lb. plantarum* 0991	−66.9
*E. coli* ATCC 8739	−0.4
*E. coli* ATCC 10536	−9.3
*S. aureus* ATCC 6538	−30.0
*S. aureus* ATCC 25923	−37.3
*K. aerogenes* ATCC 13048	−31.2
*E. faecalis* ATCC 29212	−77.8
*C. albicans* ATCC 10231	−98.5
*C. lusitaniae* ŁOCK 0004	−77.1
*C. utilis* ŁOCK 0021	−7.8
*C. pelliculosa* ŁOCK 0007	−52.5

## Supplementary Materials

Supplementary File 1
