# Supplementary material for: Combined Yeast Cultivation and Pectin Hydrolysis as an Effective Method of Producing Prebiotic Animal Feed from Sugar Beet Pulp
Source: Biomolecules. 2020 May 6;10(5):724. doi: 10.3390/biom10050724 (PMC7277867; doi:10.3390/biom10050724)
Supplement: Supplementary File 1 [file biomolecules-10-00724-s001.pdf]

Table S1. Zones of inhibited growth of lactic acid bacteria by yeast. Diameter of the inhibition zone [mm] ± standard deviation.

	Yeast strain								
**Bacteria strain**	*S. cerevisiae* Ethanol Red	*S. cerevisiae TT* ŁOCK105	*M. sinensis* 1.4 TKC.	*M. pulcherrima* NCYC747	*S. cerevisiae* Lalvin ICV K1	*S. cerevisiae* Tokay ŁOCK0204	*K. lactis* ŁOCK0028	*K. marxianus* NCYC179	*Sch. stiptis* NCYC1541
**ŁOCK0900**	0,0±0,0	0,0±0,0	0,0±0,0	0,0±0,0	0,0±0,0	0,0±0,0	0,0±0,0	0,0±0,0	0,0±0,0
**ŁOCK0912**	0,0±0,0	0,0±0,0	0,0±0,0	0,0±0,0	0,0±0,0	0,0±0,0	0,0±0,0	0,0±0,0	0,0±0,0
**ŁOCK0924**	0,0±0,0	0,0±0,0	0,0±0,0	0,0±0,0	0,0±0,0	0,0±0,0	0,0±0,0	0,0±0,0	0,0±0,0
**ŁOCK0983**	0,0±0,0	0,0±0,0	0,0±0,0	1,3±0,6	0,0±0,0	0,0±0,0	1,0±0,0	0,0±0,0	0,0±0,0
**ŁOCK0990**	0,0±0,0	0,0±0,0	0,0±0,0	0,0±0,0	0,0±0,0	0,0±0,0	0,0±0,0	0,0±0,0	0,0±0,0
**ŁOCK0991**	0,0±0,0	0,0±0,0	0,0±0,0	0,0±0,0	0,0±0,0	0,0±0,0	0,0±0,0	0,0±0,0	0,0±0,0
**ŁOCK0993**	0,0±0,0	0,0±0,0	0,0±0,0	0,0±0,0	0,0±0,0	0,0±0,0	0,0±0,0	0,0±0,0	0,0±0,0
**W3A**	0,0±0,0	0,0±0,0	0,0±0,0	0,0±0,0	0,0±0,0	0,0±0,0	0,0±0,0	0,0±0,0	0,0±0,0
**W12**	0,0±0,0	0,0±0,0	0,0±0,0	0,0±0,0	0,0±0,0	0,0±0,0	0,0±0,0	0,0±0,0	0,0±0,0
**W12A**	0,0±0,0	0,0±0,0	0,0±0,0	0,0±0,0	0,0±0,0	0,0±0,0	0,0±0,0	0,0±0,0	0,0±0,0
**W8A**	0,0±0,0	0,0±0,0	1,0±0,0	4,0±1,0	0,0±0,0	0,0±0,0	0,0±0,0	0,0±0,0	0,0±0,0
**5**	0,0±0,0	0,0±0,0	0,0±0,0	0,0±0,0	0,0±0,0	0,0±0,0	0,0±0,0	0,0±0,0	0,0±0,0
**6**	0,0±0,0	0,0±0,0	0,0±0,0	0,0±0,0	0,0±0,0	0,0±0,0	0,0±0,0	0,0±0,0	0,0±0,0
**7**	0,0±0,0	0,0±0,0	0,0±0,0	0,0±0,0	0,0±0,0	0,0±0,0	0,0±0,0	0,0±0,0	0,0±0,0
**AXD**	0,0±0,0	0,0±0,0	0,0±0,0	0,0±0,0	0,0±0,0	0,0±0,0	0,0±0,0	0,0±0,0	0,0±0,0
**AXG**	0,0±0,0	0,3±0,6	0,0±0,0	0,0±0,0	0,0±0,0	0,0±0,0	0,0±0,0	0,0±0,0	0,0±0,0
**ATCC8014**	0,0±0,0	0,0±0,0	0,0±0,0	0,0±0,0	0,0±0,0	0,0±0,0	0,0±0,0	0,0±0,0	0,0±0,0
